# Unveiling Sustainable Food Choices: How Green Attributes and Environmental Values Drive the Continuous Purchase Intentions of Organic Cocoa in Taiwan

**DOI:** 10.3390/foods15162857

**Published:** 2026-08-16

**Authors:** Li-Jie Su, Yong-Yun Cheng, Han-Shen Chen

**Affiliations:** 1 Department of Health Industry Technology Management, Chung Shan Medical University, Taichung 40201, Taiwan; lynjie1227@gmail.com; 2 In-Service Master Program of International Health Industry Management of College, Chung Shan Medical University, Taichung 40201, Taiwan; fiona263762@gmail.com; 3 Department of Medical Management, Chung Shan Medical University Hospital, Taichung 40201, Taiwan

**Keywords:** organic cocoa, green product attributes, environmental values, Theory of Planned Behavior, consumer behavior, sustainable food choice

## Abstract

As the global agri-food system faces escalating climate-related challenges, developing localized low-carbon short food supply chains (SFSCs) has become a vital strategy for sustainable agriculture. To elucidate the psychological drivers of green consumer behavior within this context, this study integrates the Stimulus–Organism–Behavior–Consequence (SOBC) framework with the Theory of Planned Behavior (TPB). The aim is to investigate the continuous purchase intentions of Taiwanese consumers for organic cocoa. Data collected via an online survey from 377 consumers with prior cocoa consumption experience were analyzed using Partial Least Squares Structural Equation Modeling (PLS-SEM). The results indicate that personal attitudes and perceived behavioral control positively drive consumption intentions, whereas subjective norms do not. This suggests that consumption behaviors rely on substantive product evaluation rather than social pressures. Additionally, green product attributes and environmental values significantly enhance TPB constructs. Importantly, consumption intentions translate positively into perceived consumer effectiveness and continuous purchase intentions. By elucidating the psychological mechanisms underlying sustainable food choices, this study highlights the value of Taiwan’s “Tree-to-Bar” model. Practically, the findings highlight potential implications for policymakers and agribusinesses regarding how environmental attributes are communicated through front-of-pack (FOP) labels that emphasize local production and low-carbon mileage. Such approaches may help make environmental information more accessible to consumers, although their effectiveness should be evaluated in future research.

## 1. Introduction

Cocoa is a vital tropical crop and core raw material for the global chocolate industry. The cocoa industry supports the livelihoods of up to 50 million people worldwide, with steady growth in production and demand [1,2]. While major African nations account for the majority of global production and exports [3], the industry faces escalating climate- and sustainability-related challenges. Against this backdrop, organic cocoa has emerged as a pivotal trajectory, with the European market showing a surging demand for certified cocoa [4]. Organic farming is widely acknowledged to balance food security with environmental conservation and mitigate multiple ecological issues [5,6]. Therefore, quantifying the behavioral drivers of organic farming consumption is paramount for addressing contemporary environmental challenges.

In subtropical regions such as Taiwan, long-term organic cultivation significantly improves soil quality and enhances agricultural sustainability [7,8]. However, importing organic food through cross-border transportation often incurs high carbon emissions, which can negate their environmental benefits [9]. Therefore, it is crucial to promote a local organic market with a small carbon footprint. As consumers’ environmental consciousness has increased, these considerations have become the primary motive for purchasing local organic foods [10]. Taiwanese consumers’ preferences for natural foods significantly shape their attitudes toward and trust in organic product labels [11,12]. Leveraging this trend, Taiwan’s local cocoa industry (e.g., Pingtung cocoa) has developed a unique “Tree-to-Bar” short supply chain model, in which cocoa is cultivated, fermented, dried, processed, and transformed into chocolate within the local production chain. This reduces reliance on long-distance transportation and strengthens traceability and connections between farmers and chocolate producers. This model serves as a critical pathway for transitioning toward premium boutique agriculture [13]. However, challenges, such as high production costs and insufficient consumer awareness, persist.

Despite the recognized importance of organic farming, empirical research on consumers’ behavioral intentions toward organic cocoa, particularly in emerging local production contexts such as Taiwan, remains limited. More importantly, existing studies on sustainable consumption have frequently examined environmental values, green product attributes, and TPB-related factors as relatively separate predictors of purchase intentions. This provides limited insight into the psychological process through which external environmental cues are internalized and subsequently translated into behavioral responses. Although previous studies have integrated the SOBC framework with TPB, the theoretical value of this integration should extend beyond simply combining two established frameworks. In this study, SOBC provides an overarching process-oriented framework for linking external environmental and product-related stimuli with subsequent consumer responses. TPB specifies the internal psychological mechanisms through which these stimuli are translated into behavioral intention. Accordingly, this study draws on the complementary perspectives of SOBC and TPB to construct a sequential process linking environmental stimuli, internal psychological evaluations, behavioral intention, and subsequent consumption-related outcomes.

Specifically, this study positions environmental values and green product attributes as environmental stimuli and conceptualizes attitudes, subjective norms, and perceived behavioral control as internal evaluative mechanisms through which these stimuli are psychologically processed. Consumption behavioral intention is subsequently positioned as the behavioral stage, and perceived consumer effectiveness and continuous purchase intention are examined as downstream consequences. This configuration extends the conventional intention-centered application of TPB by emphasizing the process through which sustainability-related stimuli are internalized before generating behavioral responses and by examining what follows the formation of behavioral intention. Accordingly, this study addresses three research questions: (1) How do environmental values and green product attributes influence consumers’ attitudes, subjective norms, and perceived behavioral control? (2) Do these psychological factors mediate the relationships between environmental values, green product attributes, and consumption behavioral intentions? (3) How does consumption behavioral intention contribute to perceived consumer effectiveness and continuous purchase intention?

This study makes several multidimensional contributions to the literature. Academically, it fills a gap in the literature by providing empirical evidence on Taiwan’s unique “smallholder premium boutique agriculture,” characterized by small-scale farming, locally produced cocoa, and value-added production targeting differentiated premium markets. This focus moves beyond predominantly Western-centric contexts. Practically, the findings provide a consumer-centric basis for government agencies, farmers’ associations, and enterprises to consider how environmental attributes are communicated through product labeling, such as front-of-pack (FOP) information and green marketing initiatives. These findings may inform the development of such strategies, although their actual effectiveness in influencing consumer responses requires further empirical validation. Finally, this study resonates with global sustainability agendas by aligning with the United Nations Sustainable Development Goals (SDGs 12, 13, and 15), offering valuable insights into the global development of short food supply chains (SFSCs) and sustainable agriculture.

## 2. Literature Review and Development of Hypotheses

While the Stimulus–Organism–Behavior–Consequence (SOBC) model is widely applied in green consumption research [14], its “Organism (O)” component primarily serves as a conceptual description. It lacks specific psychological constructs to measure internal processes. To address this limitation, this study embedded the TPB—specifically, attitudes (AT), subjective norms (SN), and perceived behavioral control (PBC)—into the SOBC’s “O” stage [15]. This theoretical integration explicitly elucidates how consumers process external stimuli (e.g., environmental values and green attributes) through cognitive and social evaluations. It equates them with concrete intentions for organic cocoa consumption.

Although TPB provides a well-established explanation of how attitudes, subjective norms, and perceived behavioral control shape behavioral intention, it offers less explicit guidance regarding how external environmental and product-related stimuli enter this psychological decision-making process. Therefore, this study employs SOBC as an overarching process-oriented framework and integrates TPB constructs to specify internal psychological mechanisms. Environmental values and green product attributes are conceptualized as external stimuli that provide environmental and product-related information, and attitudes, subjective norms, and perceived behavioral control represent consumers’ internal evaluations of these stimuli. Consumer behavioral intention is subsequently treated as a behavioral response rather than actual behavior. By contrast, perceived consumer effectiveness and continuous purchase intentions are conceptualized as downstream psychological and behavioral-intention outcomes. This integrated perspective extends TPB by linking external green consumption stimuli to the psychological mechanisms underlying behavioral intention and subsequent consumer responses, thereby providing a broader explanation of organic cocoa consumption than either framework alone.

### 2.1. Stimulus and Organism Interactions (H1 and H2)

Environmental values (EVs) and green product attributes (GPAs) are critical stimuli in green consumption decision-making. EVs reflect consumers’ altruistic concerns about ecological sustainability as core antecedents that significantly shape their AT, SN, and PBC toward eco-friendly products [16,17,18]. Furthermore, consumers’ environmental concerns and perceived green values effectively enhance their sensitivity to social norms and boost green behaviors [19,20,21,22].

Similarly, consumers base their purchasing decisions on tangible green attributes such as local production and low-carbon mileage, which minimize environmental burdens across the supply chain [23,24,25,26]. These tangible features enhance consumers’ evaluations of product eco-friendliness, fostering positive AT, stronger SN sensitivities via social cognition, and higher PBC by lowering psychological risks [27,28,29,30]. Thus, we hypothesize the following:

**H1a.**  *The EVs of organic cocoa have a significant positive effect on consumers’ SN*.

**H1b.**  *The EVs of organic cocoa have a significant positive effect on consumers’ AT*.

**H1c.**  *The EVs of organic cocoa have a significant positive effect on consumers’ PBC*.

**H2a.**  *The GPA of organic cocoa has a significant positive effect on consumers’ SN*.

**H2b.**  *The GPA of organic cocoa has a significant positive effect on consumers’ AT*.

**H2c.**  *The GPA of organic cocoa has a significant positive effect on consumers’ PBC*.

### 2.2. The Drivers of Consumption Intention (H3, H4, and H5)

In the context of organic food, consumers’ behavioral intentions are jointly determined by their AT, SN, and PBC. Specifically, strong social expectations and group values foster perceived behavioral legitimacy and serve as vital psychological determinants that drive purchase intention [31,32,33]. Similarly, positive cognitive evaluations of a product’s eco-friendliness significantly amplify consumption motivation [34,35,36,37,38]. Furthermore, consumers’ confidence in possessing adequate resources and capabilities to make green purchases is a crucial catalyst for actual behavior [39,40,41]. Thus, we hypothesize the following:

**H3.**  *Consumers’ SN has significant positive effects on their behavioral intentions to consume organic cocoa*.

**H4.**  *Consumers’ AT has significant positive effects on their behavioral intentions to consume organic cocoa*.

**H5.**  *Consumers’ PBC has significant positive effects on their behavioral intentions to consume organic cocoa*.

### 2.3. The Mediating Mechanisms (H6 and H7)

EV and cognitive awareness of products do not automatically translate into purchasing actions; they require psychological internalization [42]. The existing literature confirms that these external green stimuli must be processed by evaluating AT, SN, and behavioral capacities to identify concrete purchase intentions [43,44,45]. Tangible green attributes also have a positive indirect effect on purchase intention by cultivating green knowledge and reinforcing sensitivity to social norms [29,30]. Accordingly, we hypothesize the following:

**H6.**  *SN, AT, and PBC significantly mediate the relationship between EV and CBI*.

**H7.**  *SN, AT, and PBC significantly mediate the relationship between GPA and CBI*.

### 2.4. Consequences: Effectiveness and Continuous Purchase Intention (H8 and H9)

When consumers form strong intentions to support sustainable products, this commitment reinforces perceived consumer effectiveness (PCE), which is the conviction that their actions meaningfully alleviate socio-environmental issues [46,47,48,49]. Moreover, initial behavioral intentions are primary catalysts for future repeat purchases. Stable green intentions rooted in EVs not only determine immediate decisions but also significantly enhance consumers’ propensity for long-term patronage and continuous purchasing [50,51,52]. Thus, we hypothesize the following:

**H8.**  *Consumers’ CBI toward organic cocoa has a significant positive effect on their PCE*.

**H9.**  *Consumers’ CBI toward organic cocoa has a significant positive effect on their continuous purchase intentions*.

## 3. Methodology

### 3.1. The Research Framework

**Figure 1:**
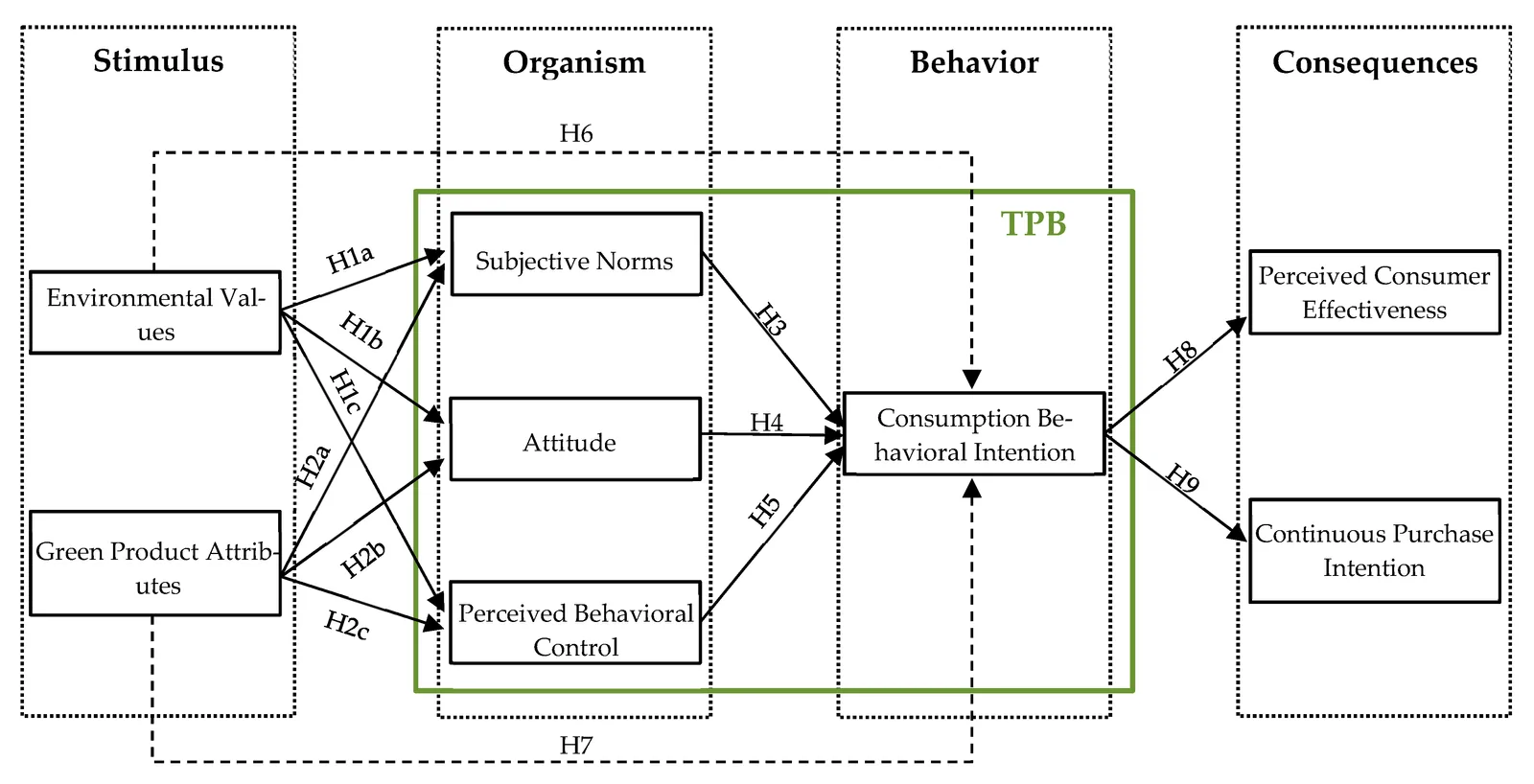
Research framework of the study.

This study is anchored in the SOBC theoretical framework and integrates the core variables of the TPB. The primary objective of this study was to investigate Taiwanese consumers’ behavioral intentions toward organic cocoa consumption, as well as their subsequent PCE and continuous purchase intentions. The research framework is illustrated in Figure 1.

### 3.2. Questionnaire Design and Measurements

To ensure the appropriateness and conceptual consistency of the measurements in the localized empirical context of Taiwan, all scales were adapted from well-established instruments in the literature. Given that the original items were developed in English, rigorous translation and back-translation procedures were used. The items were first translated into Chinese and semantically refined to align them with the cognitive framework of Taiwanese consumers regarding organic cocoa. Subsequently, academic experts were invited to assess content validity and ensure that the items accurately reflected the research constructs and eliminated any semantic ambiguity. This procedure guarantees cross-cultural measurement equivalence and enhances the scale’s reliability and validity.

To ensure that the sample accurately represented the target population, a screening question (“Have you ever purchased or consumed cocoa/organic cocoa products?”) was implemented at the beginning of the survey. Only respondents who answered “Yes” (indicating prior consumption experience) were permitted to proceed to the main survey; otherwise, they were excluded from analysis. This screening mechanism effectively eliminates invalid responses, thereby enhancing the appropriateness and robustness of the results.

The questionnaire comprised two main sections. The first section collected respondents’ demographic information, including sex, age, education level, and monthly income. The second section measured the eight core constructs using a 7-point Likert scale, ranging from 1 (“strongly disagree”) to 7 (“strongly agree”), where higher scores indicated a more positive evaluation of the respective attribute.

All measurement items were adapted from established scales in the environmental, green consumption, and organic food consumption literature. The measurement items for EV were adapted from Xie and Madni [22] and Ghazali et al. [53]. GPAs were adapted from Song et al. [26], Riva et al. [54], and Siyal et al. [55], while SNs were adapted from Xie and Madni [22], Li and Shan [45], and Zayed et al. [56] and ATs and PBC were adapted from Li and Shan [45] and Zayed et al. [56]. CBI was adapted from Li and Shan [45] and Tandon et al. [57]; PCE was adapted from Park-Poaps and Han [58]; and CPI was adapted from Tian et al. [52] and Bhutto et al. [59]. The detailed measurement items and their corresponding literature sources are presented in the measurement model assessment section (see Section 4.2).

### 3.3. Data Analysis Methods

This study employs a quantitative research approach and utilizes a structured online survey to collect empirical data. Data analyses were conducted using SPSS version 31.0 (IBM Corp., Armonk, NY, USA) and SmartPLS version 4.0 (SmartPLS GmbH, Oststeinbek, Germany). The analytical procedures encompassed the following phases.

1. Descriptive Statistics: The demographic distribution of the sample, including foundational variables such as sex, age, education level, and monthly income, was examined using SPSS 31.0.
2. Reliability and Validity Analysis: Cronbach’s α was calculated to assess the internal consistency and stability of the measurement items. Furthermore, the construct validity of the measurement model was rigorously evaluated to ensure that the scales appropriately captured the psychological dimensions targeted.
3. Structural Equation Modeling (SEM): Partial Least Squares Structural Equation Modeling (PLS-SEM) was performed using SmartPLS 4. This method was selected to test the causal relationships between the variables and the proposed hypotheses by evaluating the path coefficients, coefficients of determination (*R*^2^), predictive relevance, and out-of-sample predictive performance [60]. PLS-SEM was selected because the present study aims not only to examine theoretically hypothesized relationships but also to develop a prediction-oriented model of consumers’ behavioral intentions toward organic cocoa. The proposed model integrates environmental values and green product attributes with the psychological mechanisms of the Theory of Planned Behavior and includes multiple direct and indirect pathways. Therefore, PLS-SEM was considered appropriate for assessing the explanatory and predictive performance of the proposed structural model. In addition to assessing the measurement and structural models, the model’s out-of-sample predictive performance was evaluated using PLSpredict.

## 4. Results

### 4.1. Data Collection and Sample Profile

Data were collected via an online survey distributed through social media platforms (e.g., Facebook, Instagram, and LINE) from 11 March to 18 April 2026. A non-probability sampling approach was adopted, as participants were recruited through accessible online networks. The target population comprised consumers with prior experience of cocoa consumption. Ethical research protocols were strictly followed; all participants were informed regarding the purpose of the study and provided informed consent prior to completing the anonymous survey.

In total, 385 responses were received, of which 377 were retained as valid after screening, resulting in a valid response rate of 97.9%. Because the survey was disseminated through social media networks and the number of individuals exposed to the survey invitation could not be determined, a conventional response rate could not be calculated. As shown in Table 1, female respondents accounted for 60.2% of the sample, while male respondents accounted for 39.8%. The largest age group was 21–30 years (29.7%), followed by 41–50 years (21.0%). The sample was relatively highly educated, with 56.5% holding a college or university degree and 32.6% holding a master’s degree or above. For monthly income, the largest groups earned NT$20,000 or below (28.6%) and NT$40,001–NT$60,000 (25.7%).

To assess the sample’s fit to the broader Taiwanese population, demographic characteristics were considered against official population statistics. Taiwan’s sex ratio has been gradually declining, indicating an increasing proportion of females in the population [61]. Thus, although female respondents were relatively overrepresented in the present sample, the observed gender distribution is broadly consistent with the longer-term demographic trend. Regarding educational attainment, the Ministry of the Interior (2026) reported that, by the end of 2025, individuals aged 15 years and above with a higher education qualification accounted for 51.4% of Taiwan’s population [62]. Because educational attainment in the present sample remains higher than the general population, this should be considered when interpreting the findings, as it may influence consumers’ access to and understanding of information on organic and sustainable products.

A priori power analysis was conducted using G*Power version 3.1.4 software (Heinrich-Heine-Universität Düsseldorf, Düsseldorf, Germany) to verify the adequacy of the sample size. Based on a multiple regression model framework (F-test), the parameters were set to a significance level of *α* = 0.05, a medium effect size of *f*^2^ = 0.15, and a maximum of five predictors. The analysis yielded a statistical power (1 − *β*) of 0.999, substantially exceeding the recommended threshold of 0.80, thereby confirming that the sample size had adequate statistical power. This is consistent with the findings of Cabal-Prieto et al. (2023) [63], who noted that a moderate sample size provided acceptable detection power.

The current study obtained 377 valid responses. To rigorously assess the adequacy of the sample size, the inverse square-root approach proposed by Kock and Hadaya (2018) was applied, as this method is specifically applicable to Partial Least Squares Structural Equation Modeling [64]. Under a significance level of 0.05 and a minimum statistical power of 0.80, this approach determines the required minimum sample size based on the smallest relevant path coefficient in the structural model. The SmartPLS results showed that the smallest path coefficient among the supported hypotheses was 0.131. Based on the inverse square-root approach, a minimum sample size of approximately 361 observations was required to detect this effect. Therefore, 377 valid responses obtained in this study exceeded the estimated minimum sample size, providing an adequate sample for the PLS-SEM analysis and the subsequent assessment of hypothesized structural relationships, including mediation effects.

### 4.2. Measurement Model Assessment

The reliability and validity of the measurement model were rigorously evaluated. As shown in Table 2, Cronbach’s α values for all constructs ranged from 0.807 to 0.930. This exceeds the recommended threshold of 0.70, indicating high internal consistency. Following Purwanto and Sudargini (2021) [60], Cronbach’s α values above 0.70 are considered acceptable, while values above 0.80 indicate high reliability. Convergent validity was assessed using outer loadings, composite reliability (CR), and average variance extracted (AVE). Under ideal conditions, outer loadings should exceed 0.708, CR values should be above 0.70, and AVE values should exceed 0.50 [60]. The CR values for all constructs exceeded 0.70, and AVE values exceeded 0.50, indicating good reliability. Although one item exhibited an outer loading of 0.658, it was retained because its removal did not significantly enhance overall content validity, aligning with the recommendations of Muhamad and Zainuddin (2025) [65].

To address the potential common method bias (CMB) inherent in self-reported data, Harman’s single-factor test was performed. The first principal component accounted for 56.240% of the variance, which slightly exceeded the 50% threshold. However, recent methodological research has questioned the reliability of Harman’s single-factor test as a standalone diagnostic for CMB, noting that the variance explained by the first factor is not sufficiently sensitive to important characteristics of research design [66]. Therefore, a full collinearity assessment was conducted [67]. The full collinearity VIF values ranged from 1.441 to 4.042, remaining below the conservative threshold of 5.0 [68]. Although these results do not completely rule out the presence of CMB, they suggest that common method variance is unlikely to be the only factor accounting for the relationships observed. Nevertheless, given that all constructs were measured using self-reported responses collected from the same questionnaire, the potential influence of CMB should be acknowledged as a methodological limitation and taken into consideration when interpreting the findings.

Discriminant validity was assessed using both the Fornell–Larcker criterion and the heterotrait/monotrait (HTMT) ratio [69]. The results are presented in Table 3. Most constructs satisfied the Fornell–Larcker criterion as the square roots of the AVE exceeded their respective inter-construct correlation values. The HTMT ratio was evaluated to ensure methodological rigor. While the HTMT values for most constructs were below the stringent 0.90 threshold, the value between CBI and CPI was 0.952. Although the HTMT value between consumption behavioral intention and continuous purchase intention was 0.952, exceeding the commonly recommended threshold of 0.90, additional bootstrapping analysis was conducted to assess discriminant validity. The 95% confidence interval for the HTMT ratio ranged from 0.930 to 0.972 and did not include 1.00, providing additional statistical evidence that the two constructs remained empirically distinguishable [70]. The relatively high HTMT value nevertheless indicates that the two constructs are strongly related. Theoretically, consumption behavioral intention reflects consumers’ intention to purchase organic cocoa, whereas continuous purchase intention reflects their intention to maintain or repeat such purchasing in the future. Thus, the two constructs represent closely related but conceptually different aspects of the consumer decision process.

### 4.3. Structural Model Assessment and Predictive Relevance

The overall fit of the research model was evaluated using established model-fit indices. Following the recommendations of Hair et al. (2019) [71], the Standardized Root Mean Square Residual (SRMR) was used as the primary indicator of model fit, where a value below 0.08 indicated good fit. In this study, the SRMR value was 0.056, well below the recommended threshold, demonstrating the satisfactory overall fit of the proposed model.

Furthermore, the structural model’s explanatory power and predictive relevance were assessed using the coefficient of determination (R^2^) and the cross-validated redundancy measure (Q^2^); the results are summarized in Table 4. According to Subhaktiyasa (2024) [72], the R^2^ value measures the proportion of variance in the endogenous constructs explained by the exogenous variables, with values of 0.75, 0.50, and 0.25 corresponding to substantial, moderate, and weak explanatory powers, respectively. The findings reveal that the R^2^ values for SN (0.529), AT (0.633), PBC (0.376), CBI (0.718), PCE (0.636), and continuous purchase intention (0.783) indicate moderate to substantial explanatory power for all endogenous constructs. Additionally, the Q^2^ values for all constructs were greater than zero, ranging from 0.464 to 0.667. Notably, AT (Q^2^ = 0.667), PCE (Q^2^ = 0.662), and CBI (Q^2^ = 0.648) exhibited superior predictive accuracy. These results confirm that the structural model has predictive relevance.

The effect sizes (f^2^) of direct relationships were further assessed to evaluate the extent to which each exogenous construct contributes to its corresponding endogenous construct. Following Subhaktiyasa (2024) [72], f^2^ is used to assess the effect size of direct relationships between exogenous and endogenous variables, with values of 0.02, 0.15, and 0.35 serving as reference points for small, medium, and large effects, respectively. Most direct relationships exhibited small-to-large effect sizes, while several relationships had f^2^ values below 0.02, indicating negligible effects. The f^2^ values for all direct relationships are presented in Table 5. The presence of negligible effect sizes does not invalidate the structural model or the corresponding relationships. Rather, f^2^ values below 0.02 indicate that the respective exogenous constructs make only a limited incremental contribution to explaining variance in the corresponding endogenous constructs. Thus, these results should be interpreted in conjunction with the path coefficients and their statistical significance, rather than as evidence of model inadequacy.

Predictive assessment was conducted using the PLSpredict procedure. Following Lin and Huynh (2024) [73], Q^2^_predict values greater than zero were considered indicative of predictive relevance, while RMSE and MAE were used to evaluate prediction errors, with lower values indicating better predictive performance. The prediction errors generated by the PLS-SEM model were further compared with those of the linear model (LM) benchmark; the results are presented in Table 6. All indicators yielded positive Q^2^_predict values, ranging from 0.163 to 0.511, indicating predictive relevance. In terms of prediction errors, the PLS-SEM model produced lower RMSE values than the LM benchmark for 23 of the 27 indicators. The MAE results showed a generally similar pattern. Although several indicators exhibited higher prediction errors under PLS-SEM than the LM benchmark, particularly the PCE indicators, the overall results indicate that the proposed model demonstrates satisfactory out-of-sample predictive performance.

### 4.4. Hypothesis Testing: Direct and Mediation Effects

**Figure 2:**
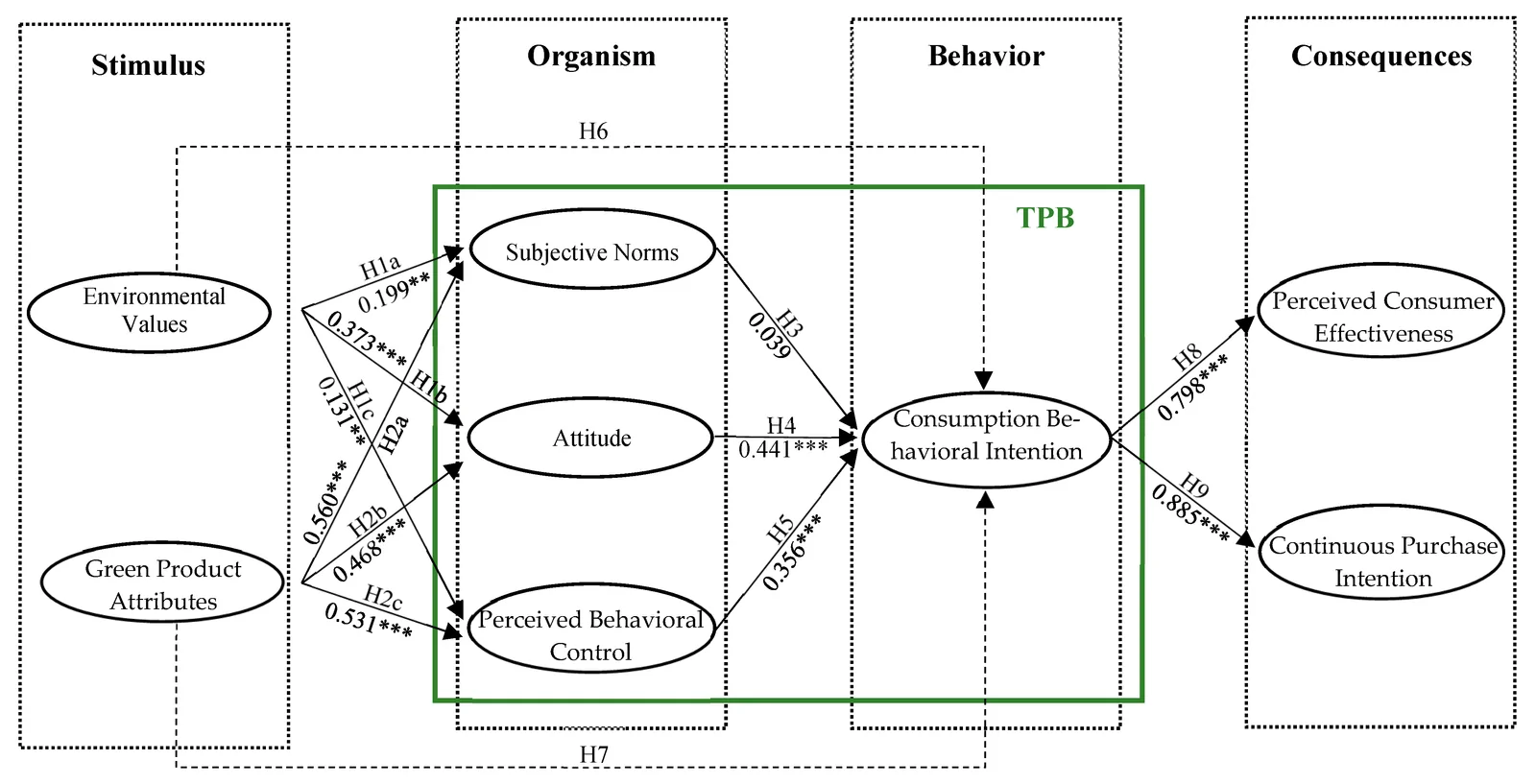
Analysis results of the structural model. Path coefficients are presented as standardized estimates (*β*). *** *p* < 0.001; ** *p* < 0.01.

Structural models and hypotheses were evaluated using PLS-SEM. Table 7 and Figure 2 summarize the complete path analysis and mediation results. For the direct effects (Panel A), EVs and GPAs significantly enhanced SNs, AT, and PBC, fully supporting H1a–c and H2a–c. Furthermore, AT and PBC exerted significant positive impacts on CBI, supporting H4 and H5. However, the effect of SN on CBI was insignificant, thus rejecting H3. Consequently, CBI positively drove perceived PCE and CPI, thereby supporting H8 and H9.

Regarding mediation effects (Panel B), the total indirect effect of GPAs on CBI was significant, supporting H7. At the specific-path level, the indirect effects of AT and PBC were significant, whereas the indirect effect of SNs was not significant. For H6, the total indirect effect of EVs on CBI was significant (total indirect effect = 0.218, *p* < 0.001). Among the specific indirect pathways, only the pathway through AT was significant, whereas the pathways through SNs and PBC were not significant. These findings indicate that AT represents a specific mediating pathway between EVs and CBI, while AT and PBC serve as significant mediating pathways between GPA and CBI.

## 5. Discussion

The findings of this study reveal that consumers’ attitudes and perceived behavioral control have significant, positive effects on their behavioral intentions to consume organic cocoa. This aligns with the results of Pacho (2020) [35] and Hasan and Suciarto (2020) [39]. In the context of organic food and green consumption, when consumers have a positive opinion of organic cocoa and perceive themselves as having adequate resources and conditions to make a purchase, their likelihood of forming a behavioral intention increases significantly. Consumers’ positive perceptions of EVs and the green attributes of organic cocoa translate into actual purchasing and support tendencies. Furthermore, PBC reflects consumers’ judgment of their ability to successfully complete a purchase, thereby serving as a critical determinant of consumption intention.

The results also indicate that SNs do not have a significant effect on CBIs toward organic cocoa. Previous studies have noted that when making purchasing decisions for organic products, consumers may not be overtly influenced by social pressure from family, friends, or significant others, which reduces the impact of SNs on purchase intention [74]. Hussin et al. (2025) [75] highlight the influence of cultural values on consumers’ motivations and decision-making patterns regarding organic food. Even in Asian societies traditionally characterized by collectivism, the rise in individualized consumption awareness has led consumers to base their purchasing decisions more on personal value cognition, product evaluation, and self-judgment rather than relying solely on external social norms or others’ opinions. This finding suggests that organic cocoa remains a niche and highly specialized agricultural product in Taiwan; consequently, the influence of SN on consumption intentions was relatively limited. This also challenges the traditional view of Asian consumers as highly susceptible to social norms; instead, it suggests emerging green consumption trends and cultural shifts.

This study incorporated variables such as EVs, GPAs, PCE, and continuous purchase intention. The results demonstrate that EVs exert a significant positive effect on SNs, AT, and PBC. This indicates that the more consumers value environmental protection, sustainable development, and green consumption principles, the higher their overall evaluation of organic cocoa, perceived capability to carry out green behaviors, and perception of significant others’ support for green consumption. This finding is consistent with Dalila et al. (2020) [16], who demonstrated that consumers with higher environmental consciousness and sustainable values are more likely to form positive attitudes, higher PBC, and stronger perceptions of social norms.

The mediation analysis revealed that the total indirect effect of EVs on CBI was significant. At the specific-path level, the indirect effect of AT was significant, whereas the indirect effects of SN and PBC were insignificant. This suggests that consumers’ environmental values may influence their consumption behavioral intentions primarily through their attitudes toward organic cocoa rather than through subjective norms or perceived behavioral control. Furthermore, the specific indirect effect of PBC was not significant, consistent with the findings of Zhao et al. (2022) [76]. Zhao et al. (2022) [76] noted that the mediating role of PBC in the influence of EVs on green purchase intentions may be constrained by factors such as product accessibility, consumption habits, or market maturity. Therefore, they may not consistently produce a significant mediating effect.

The results demonstrate that when consumers possess a higher awareness of organic cocoa attributes, such as eco-friendly carbon reduction labels, sustainable material sourcing, local production, low-carbon mileage, and green information disclosure, their SNs, AT, and PBC are all significantly and positively affected. Further comparison of the effect sizes revealed that GPAs had a highly significant impact on SNs (*p* < 0.001), AT (*p* < 0.001), and PBC (*p* < 0.001), with the most pronounced effect on SNs. This indicates that when a product features explicit green and sustainable characteristics, it fosters consumer identification with social support and green consumption values.

Furthermore, mediation analysis revealed that SNs, AT, and PBC had varying degrees of mediating effects on green GPAs and CBI. Notably, the specific indirect effects of AT and PBC were significant. This suggests that when a product possesses tangible green attributes such as local production, low-carbon mileage, eco-friendliness, and transparent green information, consumers tend to extend this positive environmental association to other dimensions of product evaluation. Consequently, consumers may be more inclined to purchase organic cocoa because these green attributes strengthen their environmental evaluations and psychological responses toward the product, thereby stimulating their purchase intentions. This phenomenon implies that, beyond enhancing consumers’ environmental identification and PBC, GPAs can also positively shape their expectations regarding overall product quality and consumption experience. This strengthens consumers’ purchase intentions and inclination for continuous support. This finding aligns with Wijekoon and Sabri’s (2021) [30] perspective, confirming that the impact of GPAs on consumption intention is not entirely direct; rather, it depends on consumers’ internal psychological cognitive processes.

The results also indicate that CBI has a significant positive effect on PCE (*p* < 0.001). When consumers develop a stronger intention to purchase and support organic cocoa, they are more inclined to believe that their consumption behavior positively impacts environmental protection, sustainable agriculture, and societal value. When engaging in green consumption behaviors, consumers gradually reinforce awareness of their personal impacts. They consider their consumption choices to have practical significance and value. This result was consistent with the findings of Hanss and Doran (2019) [47].

Finally, the findings demonstrate that CBI has a significant positive effect on continuous purchase intention (*p* < 0.001), corroborating the findings of Thanki et al. (2022) [51]. This suggests that once consumers form a stable, positive intention to consume based on EVs and GPAs, their propensity to continuously purchase and support organic cocoa products is significantly enhanced. This finding also implies that if organic cocoa products can establish a favorable initial perception of consumption and positive product experience, they will substantially contribute to cultivating consumers’ long-term patronage and continuous purchasing behaviors.

## 6. Conclusions

### 6.1. Research Findings

Based on the empirical results, this study concludes that Taiwanese consumers’ behavioral intentions toward organic cocoa are driven primarily by their AT and PBC. GPAs, including local production, low-carbon mileage, and transparent information disclosure, serve as fundamental stimuli that significantly enhance SN, AT, and PBC. Consequently, consumption intentions translate positively into PCE and continuous purchasing intentions. The influence of SNs on consumption intentions was insignificant. Although the specific indirect effects of EVs through SNs and PBC were insignificant, the total indirect effect of EVs on CBI was significant, with AT representing a significant specific mediating pathway. This indicates that consumer decision-making relies more on their individual evaluations of organic cocoa than external social pressures. Moreover, the findings suggest that environmental values do not uniformly translate into consumption behavioral intentions through all TPB pathways. Given that Taiwan’s organic cocoa market remains in its nascent stage, continued efforts by industry stakeholders and relevant public institutions may help enhance consumer awareness, product traceability, and the development of the organic cocoa market.

### 6.2. Research Contributions

Theoretically, this study extends the application of the SOBC framework by integrating it with TPB to explain how environmental values and green product attributes are translated into consumers’ behavioral responses toward organic cocoa. The findings suggest that environmental values and green product attributes do not necessarily translate directly into consumption behavioral intention; rather, their effects are shaped by consumers’ internal evaluations, including attitudes, subjective norms, and perceived behavioral control. This finding highlights the importance of considering the psychological internalization process when applying behavior-oriented theories to sustainable food consumption.

Practically, the findings highlight potential implications for chocolate brands, retailers, and policymakers. For chocolate brands, highlighting salient environmental attributes through front-of-package (FOP) labels may help make environmental information such as “Tree-to-Bar,” local smallholder sourcing, short-chain transportation, and low-carbon mileage more accessible to consumers. Retailers and online food platforms may likewise consider incorporating such environmental information into product descriptions and comparison tools. From a policy perspective, standardized environmental labeling and carbon footprint disclosure could be considered possible approaches to improving the transparency of environmental information. However, these implications are derived from the observed relationships between consumer perceptions and behavioral intentions, rather than from direct evaluations of specific marketing or policy interventions. Further research is therefore needed that empirically assesses whether these strategies can effectively influence consumer responses.

The implications may also extend beyond organic cocoa to other products with relatively high carbon footprints. For agricultural commodities such as coffee and tea, environmental attributes related to production, sourcing, processing, and transportation may similarly require clear and accessible communication to facilitate consumers’ internalization of sustainability information. For higher-carbon products such as meat and dairy, the findings suggest that environmental values alone may be insufficient to stimulate behavioral intentions. Instead, favorable attitudes, supportive subjective norms, and perceived behavioral control may be the important mechanisms that translate environmental information into decisions surrounding consumption. Nevertheless, these cross-category implications should be empirically validated because product involvement, perceived necessity, price sensitivity, and cultural meanings may differ across product categories.

### 6.3. Research Limitations

This study has several limitations. First, reliance on online non-probability sampling may overrepresent frequent Internet users, who are highly concerned with environmental sustainability, limiting the generalizability of the findings. Second, as organic cocoa remains a relatively niche product in Taiwan, some respondents might have relied on pre-existing conceptual cognition rather than long-term actual consumption experiences when completing the survey. Furthermore, self-report scales are susceptible to social desirability bias. Because all constructs were measured using self-reported items collected from the same respondents through a single questionnaire, common method bias cannot be completely ruled out. Although the full collinearity assessment did not indicate severe common method variance, the first principal component in Harman’s single-factor test accounted for 56.240% of the total variance, slightly exceeding the commonly cited 50% threshold. Given the recognized limitations of Harman’s test and the inability of the additional statistical assessment to completely exclude common method bias, this potential source of method-related bias should be considered when interpreting the findings. Future studies could employ procedural remedies such as temporal separation of measurements, multiple data sources, or longitudinal research designs to reduce and assess potential common method effects.

Methodologically, the cross-sectional design means that the empirical data indicated relatively low discriminant validity between initial consumption intention and continuous purchase intention (with an HTMT value of 0.952), suggesting an overlap in consumers’ cross-sectional perceptions. Moreover, another limitation concerns the operationalization of subjective norms. Although the construct was intended to capture consumers’ perceived social and institutional influences regarding organic cocoa consumption, some items, particularly those referring to social media influence and government policy promotion, may extend beyond the conventional TPB definition of perceived social pressure from important referents. This broader operationalization may introduce conceptual overlap between subjective norms and other forms of external influence and may have contributed to the nonsignificant relationship between subjective norms and consumption behaviors. In addition, although the significant relationship between GPA and PCE suggests that consumers’ perceptions of green attributes are associated with their perceived effectiveness in contributing to environmental outcomes, the current measurement scales do not directly capture the specific inferences that contribute to this relationship. For example, the present model does not distinguish how consumers interpret individual attributes, such as local production, low-carbon mileage, eco-friendliness, and transparent green information, when forming their perceptions of consumer effectiveness. Finally, the absence of actual product testing, such as data on taste or price variations, restrains the full reflection of real-world purchase decision-making.

### 6.4. Suggestions for Future Research

Future research should adopt longitudinal designs to track the dynamic evolution of public cognition, attitudes, and purchasing behavior toward organic agricultural products over time. Subsequent studies should integrate additional variables such as health consciousness, brand image, price sensitivity, and food safety trust. Methodologically, employing Choice Experiments and the Contingent Valuation Method to estimate consumers’ willingness to accept different product attributes and price premiums could result in a more comprehensive behavioral model. Additionally, utilizing qualitative in-depth interviews or focus groups could supplement quantitative data, providing a holistic understanding of the market context. Finally, future research could further elucidate the differential effects of distinct green attributes, such as fair trade, local production, and carbon footprint labels, by expanding the measurement scales. This can capture the specific inferences consumers derive from these attributes and how such inferences contribute to perceived consumer effectiveness and subsequent behavioral intentions.

## Figures and Tables

**Table 1:** Demographic profile of the respondents (*n* = 377).

Items	Categories	Frequency (N)	Percentage (%)
Gender	Male	150	39.8%
	Female	227	60.2%
Age	20 years old or younger	38	10.1%
	21–30 years old	112	29.7%
	31–40 years old	66	17.5%
	41–50 years old	79	21.0%
	51–60 years old	62	16.4%
	61 years old or older	20	5.3%
Education	Junior high school or below	5	1.3%
	Senior high/Vocational school	36	9.5%
	College/University	213	56.5%
	Master’s degree or higher	123	32.6%
Monthly Income (NT$)	$20,000 or less	108	28.6%
	$20,001–$40,000	83	22.0%
	$40,001–$60,000	97	25.7%
	$60,001–$80,000	36	9.5%
	$80,001 or more	53	14.1%

**Table 2:** Reliability and validity analysis of the measurement model.

Construct	Items	Standardized Factor Loading	VIF	Cronbach’s α	CR	AVE	Sources
Environmental Values (EV)	EV1: I believe that organic cocoa is more eco-friendly than conventional cocoa.	0.862	2.698	0.894	0.922	0.703	[22,53]
	EV2: I believe that organic cocoa can enhance environmental performance.	0.862	2.621				
	EV3: I believe that organic cocoa has a higher added value.	0.802	1.866				
	EV4: I believe that organic cocoa can reduce the emission, diffusion, and generation of harmful substances.	0.827	2.200				
	EV5: I believe that organic cocoa can maximize the sustainable use of renewable resources.	0.839	2.216				
Green Product Attributes (GPA)	GPA1: I prefer locally grown organic cocoa products.	0.836	2.365	0.903	0.928	0.721	[26,54,55]
	GPA2: I prefer organic cocoa products with low-carbon labels.	0.857	2.688				
	GPA3: I believe the eco-friendly characteristics of organic cocoa products meet my expectations.	0.881	2.925				
	GPA4: I prefer organic cocoa products because they are environmentally friendly.	0.868	2.681				
	GPA5: I believe that local organic cocoa products possess carbon-reducing characteristics.	0.800	1.963				
Subjective Norms (SN)	SN1: Purchasing organic cocoa products is encouraged in my social circle.	0.833	2.520	0.895	0.923	0.705	[22,45,56]
	SN2: Most people who are important to me would choose to buy organic cocoa products.	0.838	2.580				
	SN3: Information shared on social media influences my decision to purchase organic cocoa products.	0.818	2.137				
	SN4: The public generally believes that purchasing organic cocoa benefits social development.	0.849	2.471				
	SN5: The promotion of government policies would encourage me to buy organic cocoa products.	0.859	2.510				
Attitude (AT)	AT1: I like organic cocoa products.	0.870	2.763	0.930	0.948	0.785	[45,56]
	AT2: I think buying organic cocoa products is a good idea.	0.916	3.998				
	AT3: I think buying organic cocoa products is a wise choice.	0.881	3.001				
	AT4: I think buying organic cocoa products is important.	0.871	2.742				
	AT5: I think buying organic cocoa products is beneficial.	0.890	3.204				
Perceived Behavioral Control (PBC)	PBC1: I feel that it is easy to purchase organic cocoa products.	0.795	2.417	0.807	0.872	0.633	[45,56]
	PBC2: I have the resources and time to purchase organic cocoa products.	0.846	2.715				
	PBC3: If I want to, I can purchase organic cocoa products.	0.867	1.973				
	PBC4: Whether to buy organic cocoa products is entirely up to my own will.	0.658	1.441				
Consumption Behavioral Intention (CBI)	CBI1: I am willing to purchase organic cocoa products.	0.876	3.078	0.925	0.944	0.772	[45,57]
	CBI2: I intend to purchase organic cocoa products.	0.885	3.264				
	CBI3: I am willing to pay a premium for organic cocoa products.	0.868	3.176				
	CBI4: I am willing to buy organic cocoa products at a price higher than that of conventional cocoa.	0.901	4.042				
	CBI5: Even if conventional cocoa products were available, I would still choose to buy organic cocoa products.	0.863	2.758				
Perceived Consumer Effectiveness (PCE)	PCE1: I believe that buying organic cocoa products to protect the environment is worthwhile.	0.883	2.702	0.921	0.944	0.808	[58]
	PCE2: I believe that buying organic cocoa products helps improve environmental issues.	0.905	3.275				
	PCE3: I believe that choosing organic cocoa products allows me to protect the environment.	0.919	3.643				
	PCE4: I believe that purchasing organic cocoa products can positively impact the social environment.	0.888	2.979				
Continuous Purchase Intention (CPI)	CPI1: I will continue to buy organic cocoa products.	0.909	3.199	0.928	0.949	0.823	[52,59]
	CPI2: I intend to recommend purchasing organic cocoa products to my relatives and friends.	0.896	3.056				
	CPI3: I expect to buy organic cocoa products in the future.	0.904	3.230				
	CPI4: I plan to buy organic cocoa products in the future.	0.919	3.680				

Note: CR = Composite Reliability; AVE = Average Variance Extracted; VIF = Variance Inflation Factor. All standardized factor loadings are significant at *p* < 0.001.

**Table 3:** Discriminant validity: Fornell–Larcker criterion and HTMT ratio.

Construct	1. EV	2. GPA	3. SN	4. AT	5. PBC	6. CBI	7. PCE	8. CPI
1. EV	**0.839**	0.874	0.710	0.812	0.637	0.646	0.794	0.665
2. GPA	0.787 **	**0.849**	0.794	0.830	0.733	0.769	0.816	0.774
3. SN	0.640 **	0.717 **	**0.840**	0.800	0.776	0.737	0.737	0.737
4. AT	0.741 **	0.761 **	0.734 **	**0.886**	0.794	0.845	0.884	0.842
5. PBC	0.549 **	0.634 **	0.668 **	0.698 **	**0.796**	0.857	0.800	0.850
6. CBI	0.591 **	0.704 **	0.673 **	0.788 **	0.756 **	**0.879**	0.861	0.952
7. PCE	0.721 **	0.745 **	0.676 **	0.819 **	0.700 **	0.798 **	**0.899**	**0.833**
8. CPI	0.608 **	0.710 **	0.707 **	0.784 **	0.750 **	0.885 **	0.772 **	**0.907**

Note 1: Bold diagonal elements represent the square root of the AVE. The lower left off-diagonal elements are the inter-construct correlations (Fornell–Larcker criterion). The upper right italicized elements are the HTMT ratios. Note 2: ** *p* < 0.01. Construct Abbreviations: EVs = Environmental Values; GPAs = Green Product Attributes; SNs = Subjective Norms; AT = Attitude; PBC = Perceived Behavioral Control; CBI = Consumption Behavioral Intention; PCE = Perceived Consumer Effectiveness; CPI = Continuous Purchase Intention.

**Table 4:** Explanatory power (*R*^2^) and predictive relevance (*Q*^2^) assessments.

Construct	R^2^	Adjusted R^2^	Q^2^
Environmental Values (EVs)	-	-	0.548
Green Product Attributes (GPAs)	-	-	0.574
Subjective Norms (SNs)	0.529	0.527	0.552
Attitude (AT)	0.633	0.631	0.667
Perceived Behavioral Control (PBC)	0.376	0.373	0.464
Consumption Behavioral Intention (CBI)	0.718	0.716	0.648
Perceived Consumer Effectiveness (PCE)	0.636	0.635	0.662
Continuous Purchase Intention (CPI)	0.783	0.783	0.552

Note: EV and GPA are exogenous constructs and therefore do not have R^2^ values.

**Table 5:** Effect sizes (*f*^2^) of direct relationships.

Hypothesis/Path	f^2^
H1a: EV → SN	0.032
H1b: EV → AT	0.144
H1c: EV → PBC	0.011
H2a: GPA → SN	0.254
H2b: GPA → AT	0.227
H2c: GPA → PBC	0.181
H3: SN → CBI	0.002
H4: AT → CBI	0.194
H5: PBC → CBI	0.203
H8: CBI → PCE	1.747
H9: CBI → CPI	3.611

**Table 6:** Predictive power.

Items	Q^2^_Predict	PLS-SEM_RMSE	PLS-SEM_MAE	LM_RMSE	LM_MAE
SN1	0.332	1.086	0.846	1.104	0.862
SN2	0.314	1.225	0.957	1.230	0.958
SN3	0.294	1.128	0.863	1.157	0.888
SN4	0.411	0.968	0.733	0.984	0.742
SN5	0.466	0.960	0.736	0.972	0.745
AT1	0.475	0.903	0.673	0.914	0.681
AT2	0.508	0.853	0.619	0.861	0.625
AT3	0.471	0.888	0.671	0.904	0.678
AT4	0.511	0.959	0.706	0.981	0.714
AT5	0.481	0.891	0.646	0.904	0.656
PBC1	0.163	1.362	1.087	1.365	1.089
PBC2	0.218	1.326	1.042	1.335	1.054
PBC3	0.397	1.016	0.754	1.029	0.754
PBC4	0.199	1.045	0.809	1.059	0.797
CBI1	0.405	1.000	0.713	1.020	0.732
CBI2	0.389	1.066	0.796	1.080	0.811
CBI3	0.325	1.253	0.936	1.259	0.947
CBI4	0.387	1.181	0.867	1.195	0.883
CBI5	0.382	1.100	0.815	1.098	0.807
PCE1	0.427	0.894	0.688	0.867	0.671
PCE2	0.407	0.970	0.729	0.933	0.682
PCE3	0.459	0.930	0.710	0.887	0.648
PCE4	0.428	0.921	0.693	0.890	0.658
CPI1	0.456	1.027	0.764	1.028	0.758
CPI2	0.374	1.150	0.881	1.157	0.891
CPI3	0.400	1.102	0.845	1.110	0.850
CPI4	0.395	1.161	0.892	1.175	0.893

**Table 7:** Results of hypothesis testing (direct and mediation effects).

Hypothesis/Path	Std. Beta/Effect	CI [2.5%, 97.5%]	*t*-Value	*p*-Value	Result
**Panel A: Direct Effects**					
H1a: EV → SN	0.199	[0.069, 0.327]	3.054	**	Supported
H1b: EV → AT	0.373	[0.265, 0.477]	6.847	***	Supported
H1c: EV → PBC	0.131	[−0.000, 0.258]	1.989	**	Supported
H2a: GPA → SN	0.560	[0.440, 0.683]	8.998	***	Supported
H2b: GPA → AT	0.468	[0.360, 0.574]	8.634	***	Supported
H2c: GPA → PBC	0.531	[0.402, 0.654]	8.382	***	Supported
H3: SN → CBI	0.039	[−0.051, 0.152]	0.751	0.453	Not supported
H4: AT → CBI	0.441	[0.278, 0.567]	5.947	***	Supported
H5: PBC → CBI	0.356	[0.261, 0.447]	7.453	***	Supported
H8: CBI → PCE	0.798	[0.750, 0.840]	34.735	***	Supported
H9: CBI → CPI	0.885	[0.859, 0.907]	73.294	***	Supported
**Panel B: Mediation Effects**	**Specific Indirect Effect**	**CI** **[2.5%, 97.5%]**	**Total Indirect Effect**	***p*** **-value** **(Total Indirect)**	**Overall Result**
H6: EV → (SN, AT, PBC) → CBI			0.218	***	Supported
--Specific indirect via SN	0.005	[−0.012, 0.031]		0.647	
--Specific indirect via AT	0.165	[0.097, 0.229]		***	
--Specific indirect via PBC	0.027	[−0.000, 0.094]		0.288	
H7: GPA → (SN, AT, PBC) → CBI			0.417	***	Supported
--Specific indirect via SN	0.013	[−0.029, 0.090]		0.646	
--Specific indirect via AT	0.206	[0.125, 0.289]		***	
--Specific indirect via PBC	0.208	[0.125, 0.262]		***	

Note: *** *p* < 0.001; ** *p* < 0.01. EVs = Environmental Values; GPAs = Green Product Attributes; SNs = Subjective Norms; AT = Attitude; PBC = Perceived Behavioral Control; CBI = Consumption Behavioral Intention; PCE = Perceived Consumer Effectiveness; CPI = Continuous Purchase Intention.

## Data Availability

The data supporting the findings of this study are not publicly available because the dataset contains participant responses collected under conditions of confidentiality and is subject to privacy and data-protection considerations. No directly identifiable personal information was collected during the survey. However, the data are available from the corresponding author, H.-S.C., upon reasonable request, and with respect to the aforementioned constraints.
